# Supplementary material for: A halo of reduced dinoflagellate abundances in and around eelgrass beds
Source: PeerJ. 2020 Apr 7;8:e8869. doi: 10.7717/peerj.8869 (PMC7147434; doi:10.7717/peerj.8869)

Supplementary tables and figures

Figure S1: Schematic of transects from contiguous eelgrass beds (Eg, dark green) to bare substrate (Ba, brown). Distance from the edge of the contiguous eelgrass beds are given in meters; sites outside of the contiguous beds have an average shoot density of less than three shoots per square meter and the bare substrate site is devoid of eelgrass.

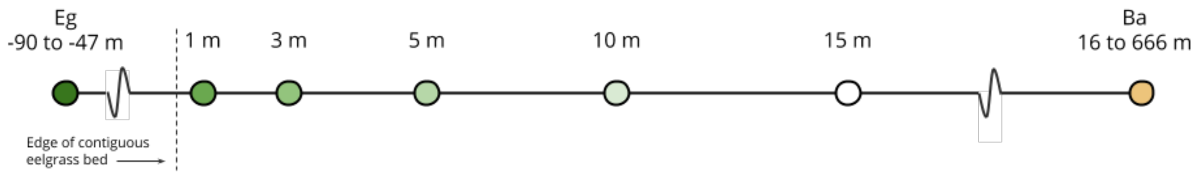

Table S1: Sample information. For each site, Case Inlet (CI), Port Gamble (PG), Nisqually Reach (NR), Skokomish (SK), and Willapa Bay (WB), approximate transect positions are recorded, as well as latitude, longitude, and the approximate geographic distance of each sample from the eelgrass bed edge, calculated from coordinates. Negative distances indicate samples within the eelgrass bed itself. Columns named May, July, and August list the number of technical replicates passing quality control measures of three sequenced from each bottle of water. NA indicates samples that were not gathered, and asterisks indicate samples for which three technical replicates were sequenced on two separate MiSeq runs to characterize the importance of sequencing run in explaining variation among samples.

Site	Position	long	lat	Distance	May	July	August
CI	Eelgrass	-122.79645	47.358439	-47	3	6*	3
CI	Along 1	-122.79584	47.358455	1	3	3	3
CI	Along 3	-122.796038	47.358565	3	3	3	3
CI	Along 6	-122.795971	47.358551	6	3	3	2
CI	Along 10	-122.795894	47.358481	10	3	3	2
CI	Along 15	-122.795817	47.358436	15	1	3	2
CI	Bare	-122.79576	47.357937	57	3	3	3
NR	Eelgrass	-122.726752	47.101926	NA	3	3	2
NR	Bare	-122.726386	47.101713	NA	3	3	3
PG	Eelgrass	-122.58292	47.847983	-80	3	3	3
PG	Along 1	-122.583221	47.84866	1	3	3	2
PG	Along 3	-122.583157	47.848705	3	3	2	2
PG	Along 6	-122.583222	47.848725	6	3	3	0
PG	Along 10	-122.583278	47.848756	10	3	3	3
PG	Along 15	-122.583258	47.848781	15	3	3	3
PG	Bare	-122.58383	47.842676	666	3	6*	3
SK	Eelgrass	-123.156623	47.354332	-52	3	3	3
SK	Along 1	-123.157147	47.354626	1	NA	3	3
SK	Along 3	-123.157132	47.354585	3	NA	3	2
SK	Along 6	-123.157116	47.354634	6	NA	3	3
SK	Along 10	-123.157162	47.354644	10	NA	3	3
SK	Along 15	-123.157185	47.354733	15	NA	3	2
SK	Bare	-123.157314	47.35502	45	3	3	3
WB	Eelgrass	-124.02619	46.495137	-90	3	3	NA
WB	Along 1	-124.02622	46.494334	1	3	3	2
WB	Along 3	-124.02627	46.494347	3	3	3	3
WB	Along 6	-124.02626	46.494425	6	3	3	3
WB	Along 10	-124.02624	46.494437	10	3	3	3
WB	Along 15	-124.02619	46.494479	15	3	3	3
WB	Bare	-124.026136	46.494479	16	3	3	3

Figure S2: Hierarchical clustering from transect Bray-Curtis distance matrices in which three technical replicates were sequenced on two different runs. Names of technical replicates contain sample information separated by '_' as follows: Site abbreviation, position abbreviation, transect distance, month, replicate, and sequencing run. Note that all replicates from Miseq run 2 (r2) cluster with the corresponding replicates from Miseq run 1 (r1) for both Port Gamble July and Case Inlet July transects.

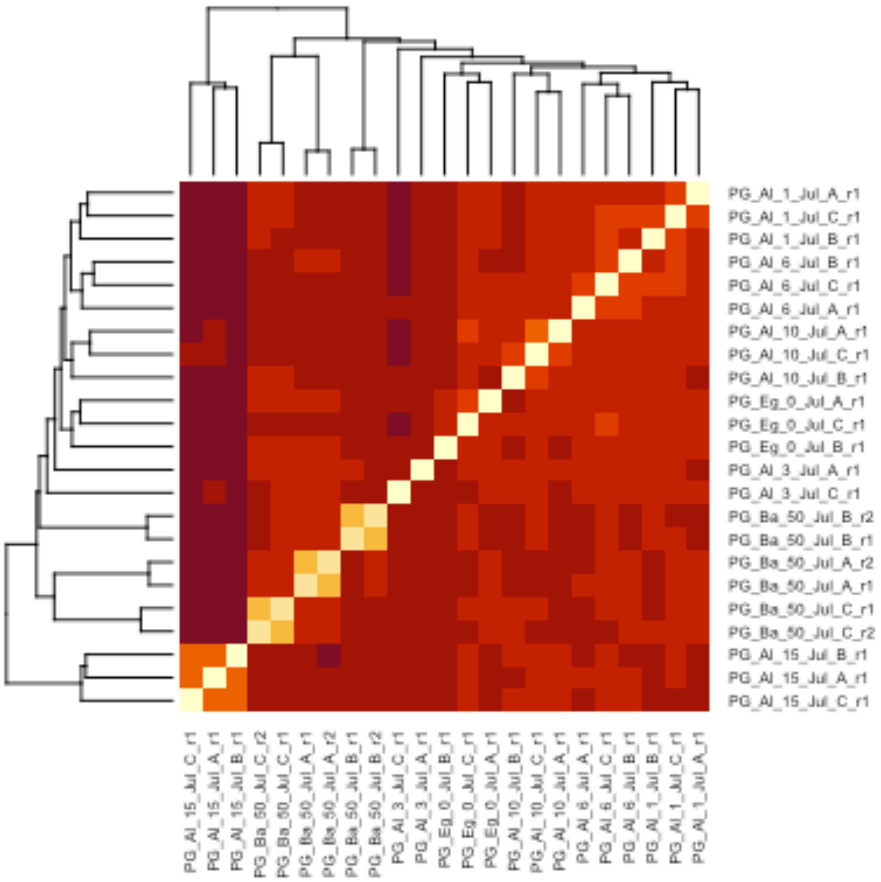

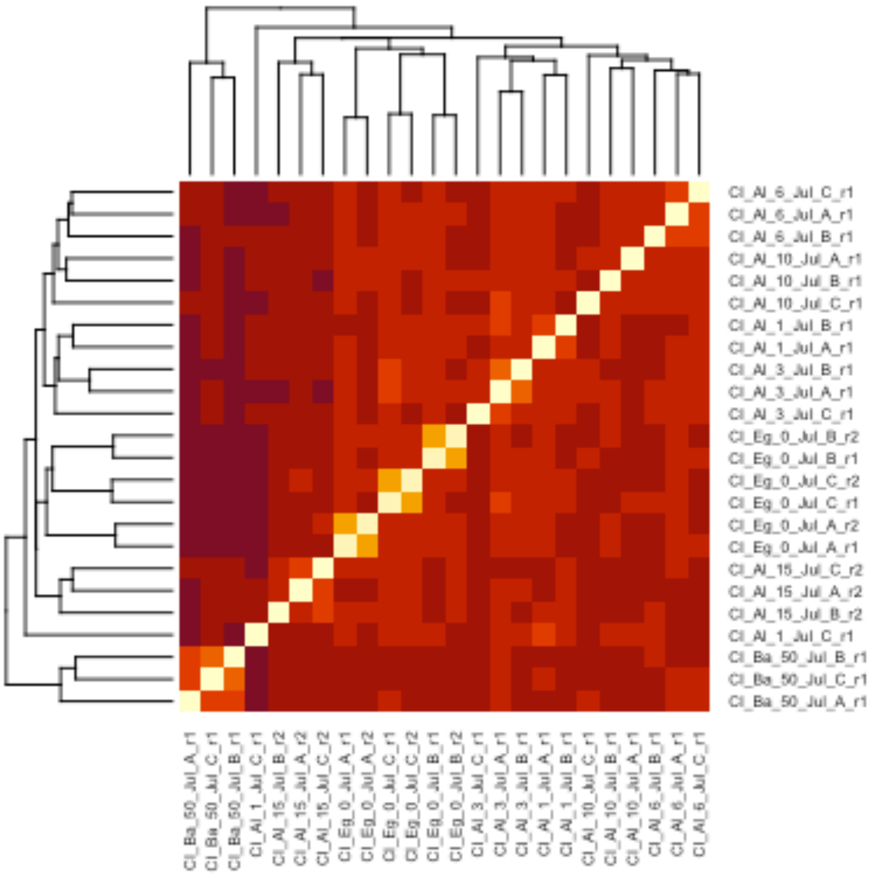

Cl_Al_6_Jul_C_r1
Cl_Al_6_Jul_A_r1
Cl_Al_6_Jul_B_r1
Cl_Al_10_Jul_A_r1
Cl_Al_10_Jul_B_r1
Cl_Al_10_Jul_C_r1
Cl_Al_1_Jul_B_r1
Cl_Al_1_Jul_A_r1
Cl_Al_3_Jul_B_r1
Cl_Al_3_Jul_A_r1
Cl_Al_3_Jul_C_r1
Cl_Eg_0_Jul_B_r2
Cl_Eg_0_Jul_B_r1
Cl_Eg_0_Jul_C_r2
Cl_Eg_0_Jul_C_r1
Cl_Eg_0_Jul_A_r2
Cl_Eg_0_Jul_A_r1
Cl_Al_15_Jul_C_r2
Cl_Al_15_Jul_A_r2
Cl_Al_15_Jul_B_r2
Cl_Al_1_Jul_C_r1
Cl_Ba_50_Jul_B_r1
Cl_Ba_50_Jul_C_r1
Cl_Ba_50_Jul_A_r1

Figure S3: High- and low-abundance transects generated with kmeans unsupervised machine learning. Black points indicate high-abundance transects; white points indicate low-abundance transects.

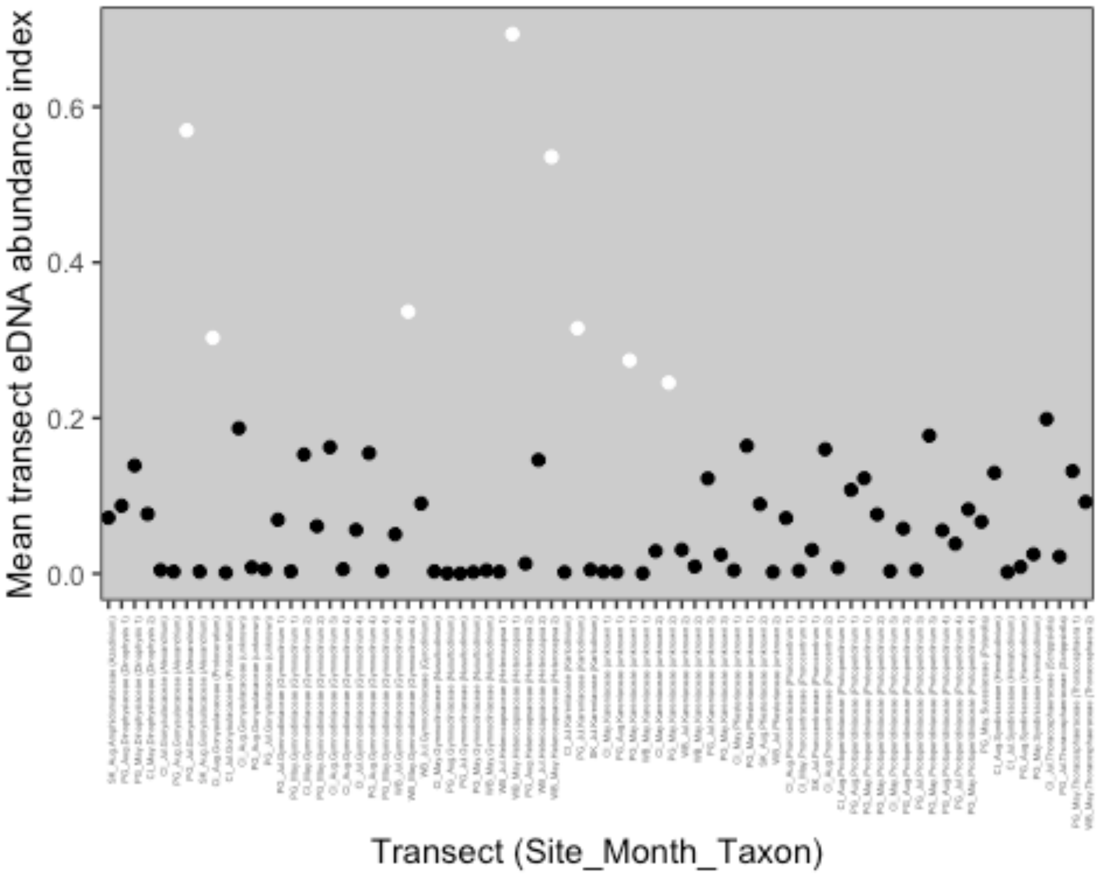

Figure S4: eDNA indices from each technical replicate of biological samples for dinoflagellates with a high-abundance transect, plotted across all months and sites. The single sample taken above eelgrass (located 47-90 meters inside the edge of the contiguous beds) is shown in green (Eg), alongshore transect samples are shown in white and labeled with distance from the contiguous eelgrass bed in meters, and the single sample taken above bare substrate (located 16-670m outside the edge of the contiguous beds) is shown in brown (Ba) Site abbreviations are as follows: CI (Case Inlet), PG (Port Gamble), SK (Skokomish), WB (Willapa Bay).

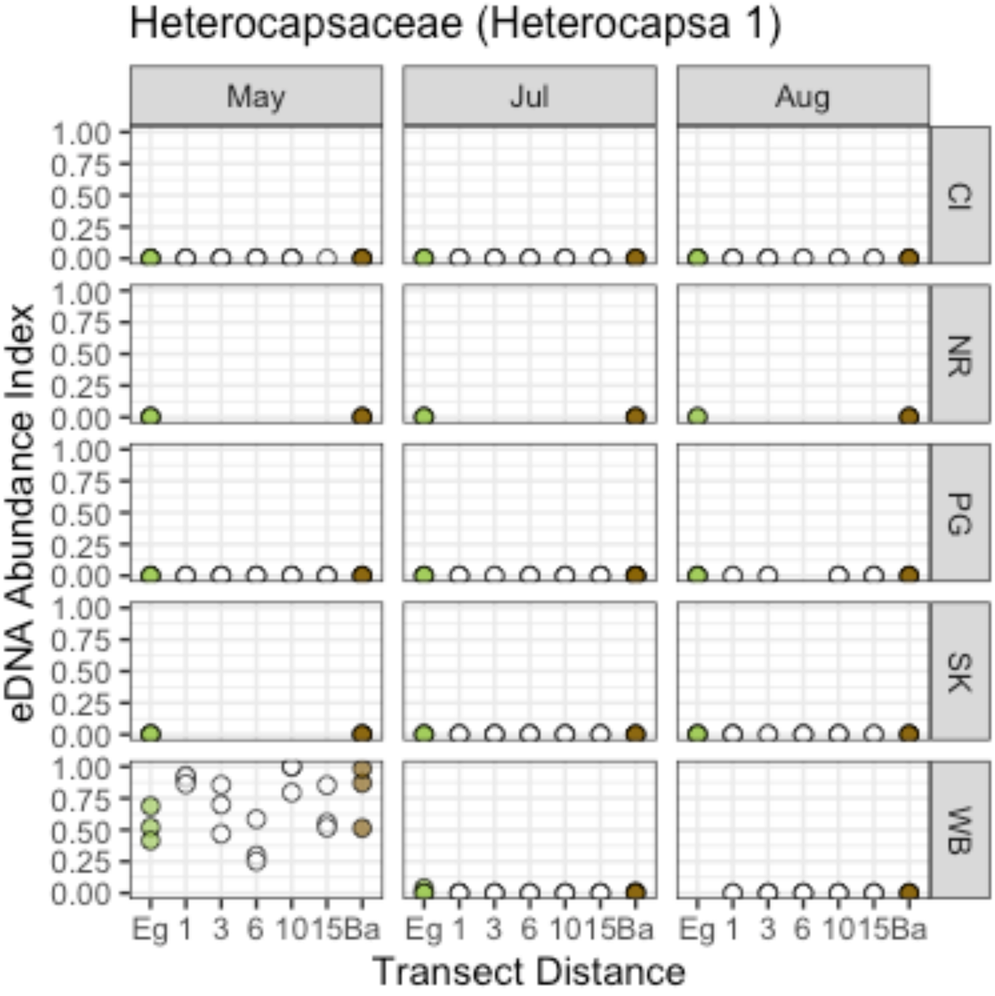

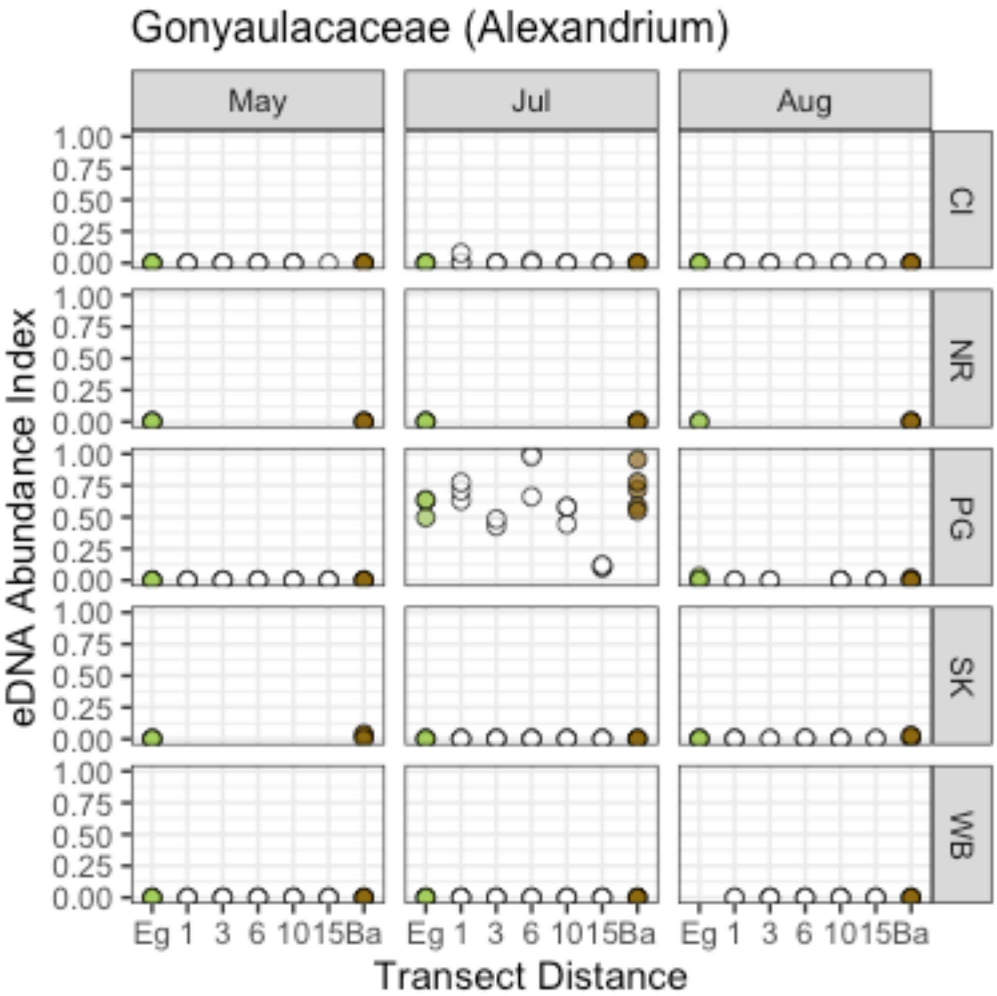

Gonyaulacaceae (Alexandrium)
May
Jul
Aug
CI
NR
PG
SK
WB
1.00
0.75
0.50
0.25
0.00
eDNA Abundance Index
Eg 1 3 6 10 15 Ba
Transect Distance

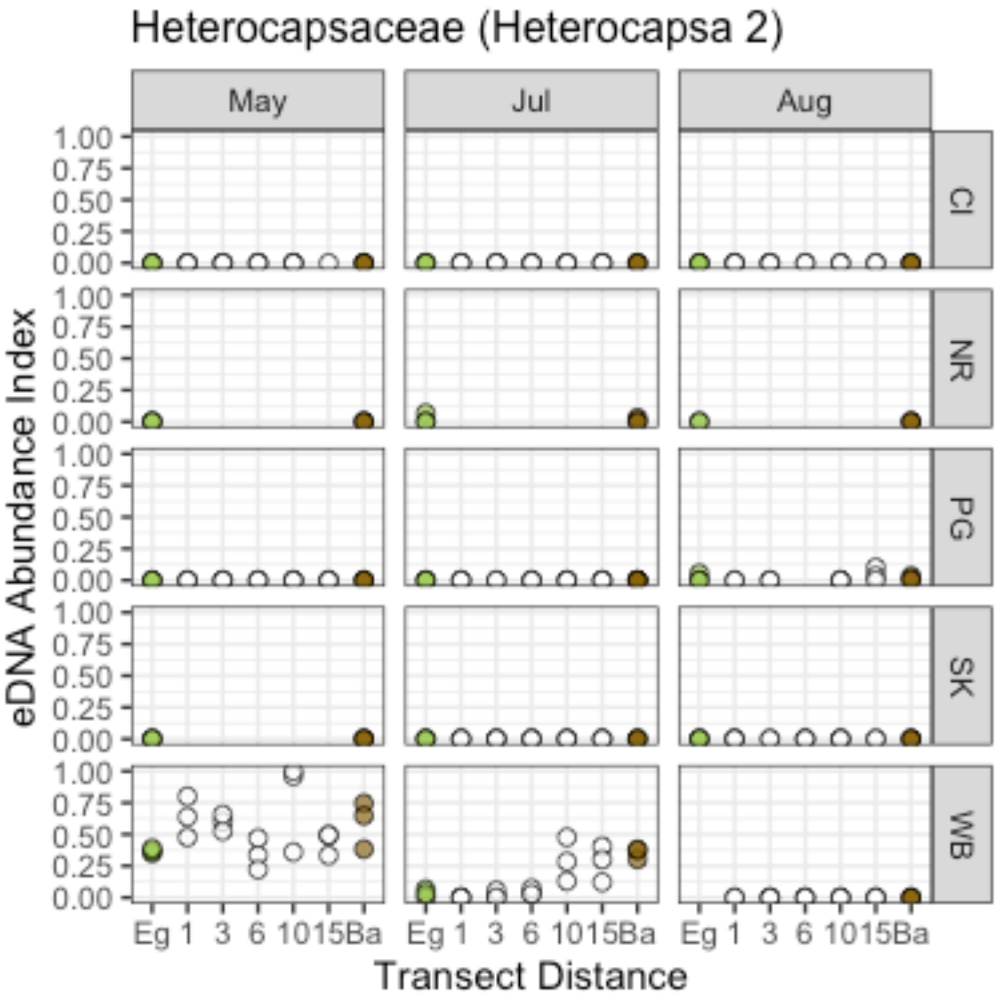

Heterocapsaceae (Heterocapsa 2)
May
Jul
Aug
CI
NR
PG
SK
WB
1.00
0.75
0.50
0.25
0.00
eDNA Abundance Index
Eg 1 3 6 10 15 Ba
Transect Distance

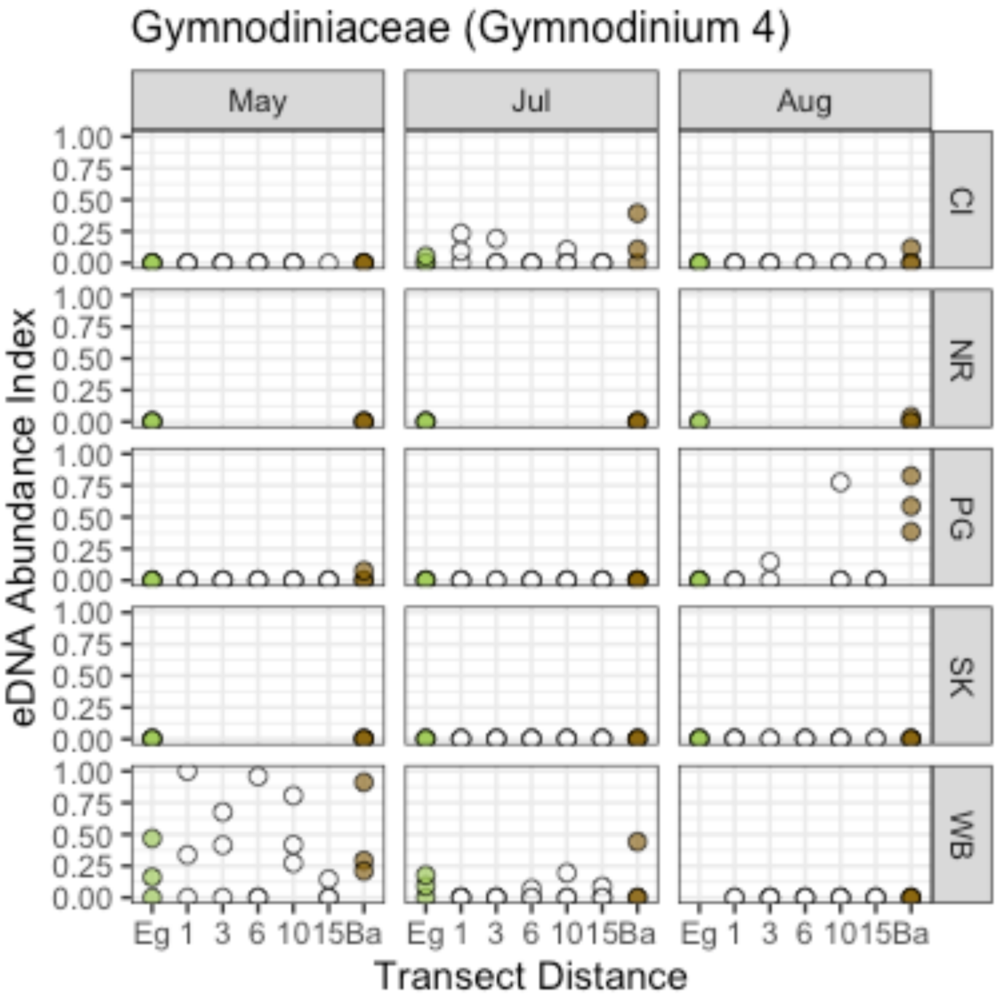

Gymnodiniaceae (Gymnodinium 4)
May
Jul
Aug
CI
NR
PG
SK
WB
1.00
0.75
0.50
0.25
0.00
eDNA Abundance Index
Eg 1 3 6 10 15 Ba
Transect Distance

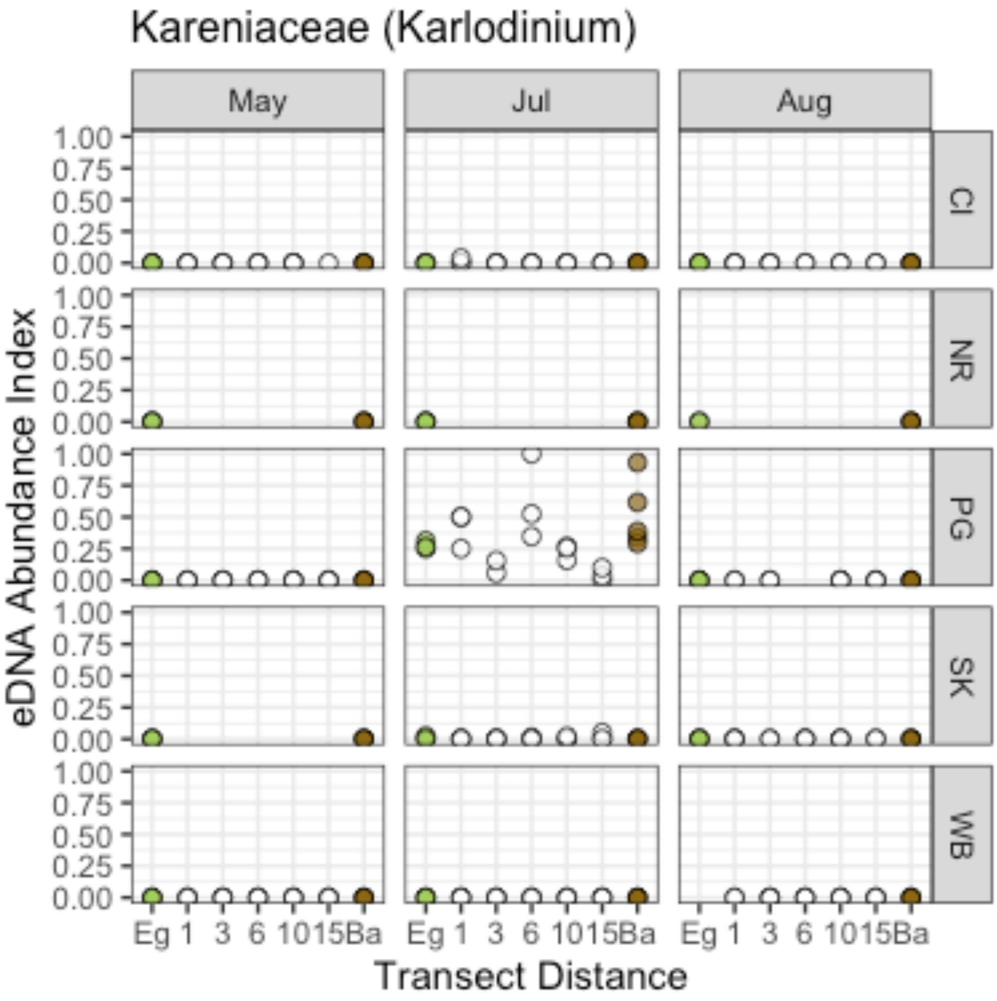
Kareniaceae (Karlodinium)
May
Jul
Aug
CI
NR
PG
SK
WB
1.00
0.75
0.50
0.25
0.00
eDNA Abundance Index
Eg 1 3 6 10 15 Ba
Transect Distance

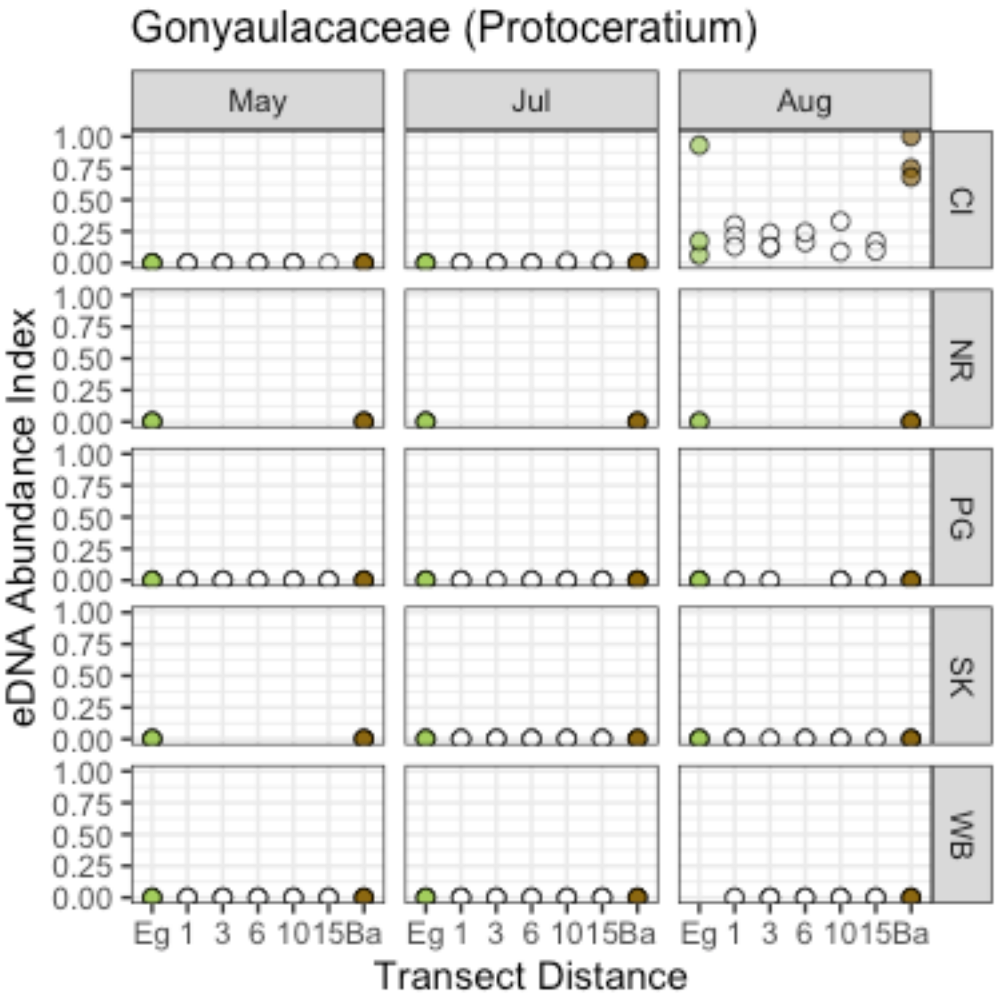

Gonyaulacaceae (Protoceratium)
May
Jul
Aug
CI
NR
PG
SK
WB
eDNA Abundance Index
1.00
0.75
0.50
0.25
0.00
Eg 1 3 6 10 15 Ba
Transect Distance

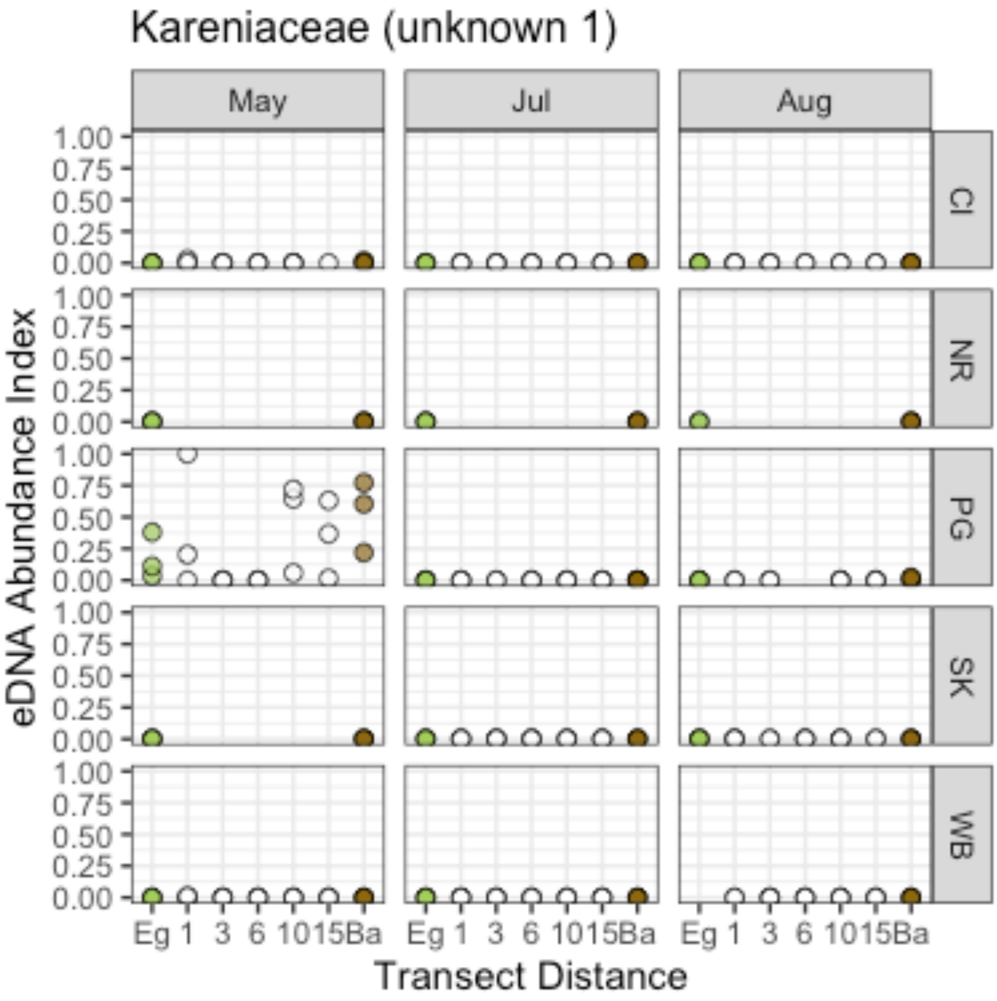
Kareniaceae (unknown 1)
May
Jul
Aug
CI
NR
PG
SK
WB
eDNA Abundance Index
1.00
0.75
0.50
0.25
0.00
Eg 1 3 6 10 15 Ba
Transect Distance

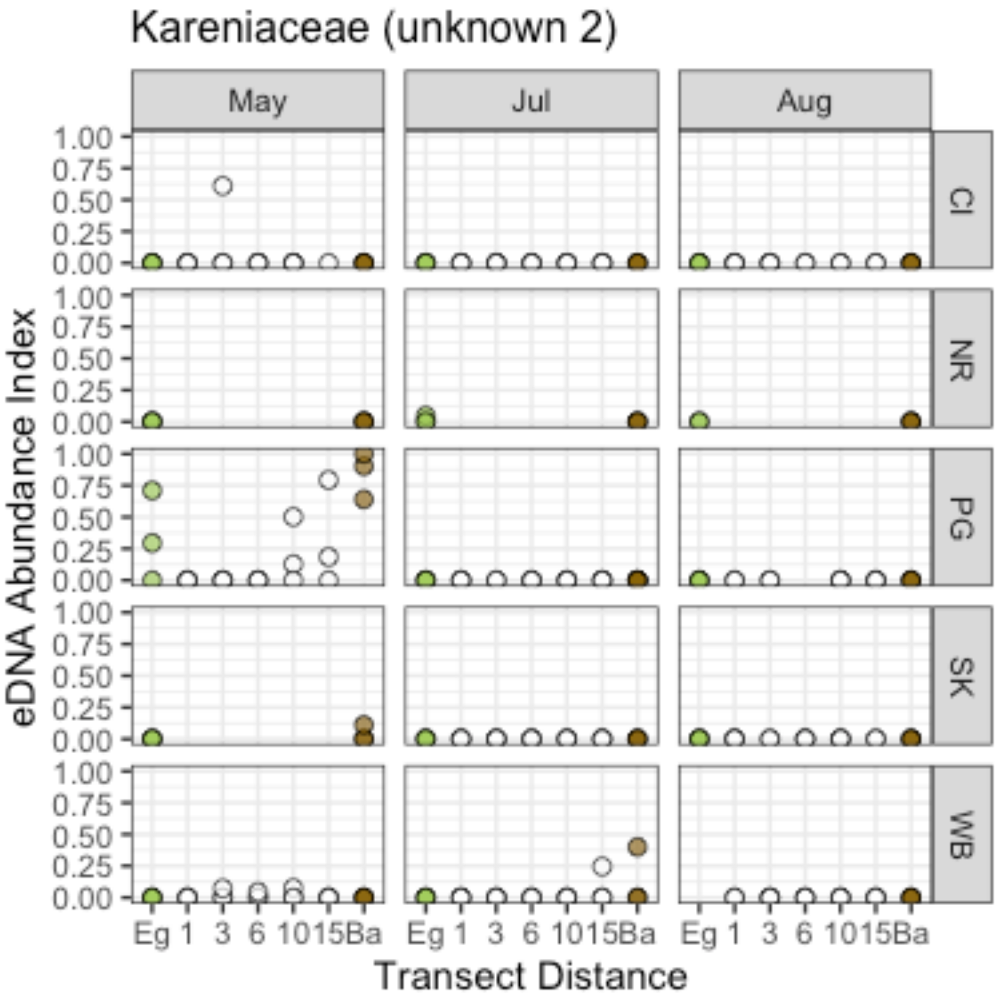

Kareniaceae (unknown 2)
May
Jul
Aug
CI
NR
PG
SK
WB
1.00
0.75
0.50
0.25
0.00
eDNA Abundance Index
Eg 1 3 6 10 15 Ba
Transect Distance

Figure S5: Phylogeny of dinoflagellate sequences from high-abundance transects. Individual sequences are named with Family_Genus information (when known).

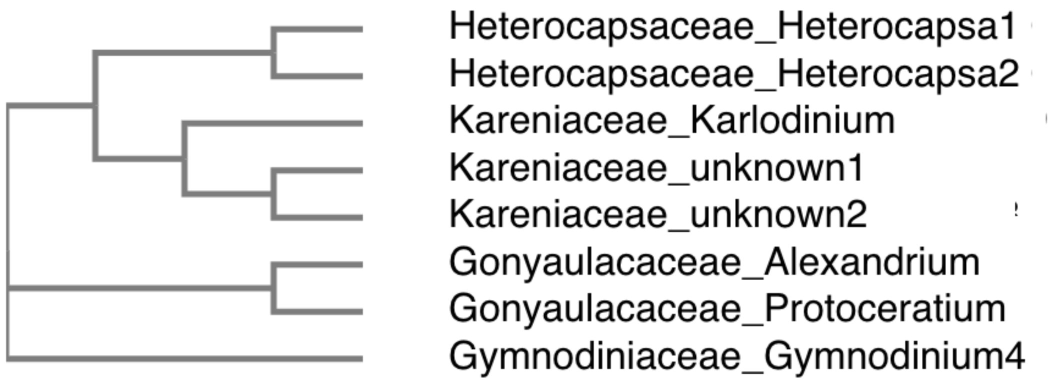

Figure S6: Bray-Curtis dissimilarity between eDNA communities surveyed at adjacent points along each full transect (all sites and months). Shading of violins indicates median spatial distance between communities (dark = closer, light = more distant).

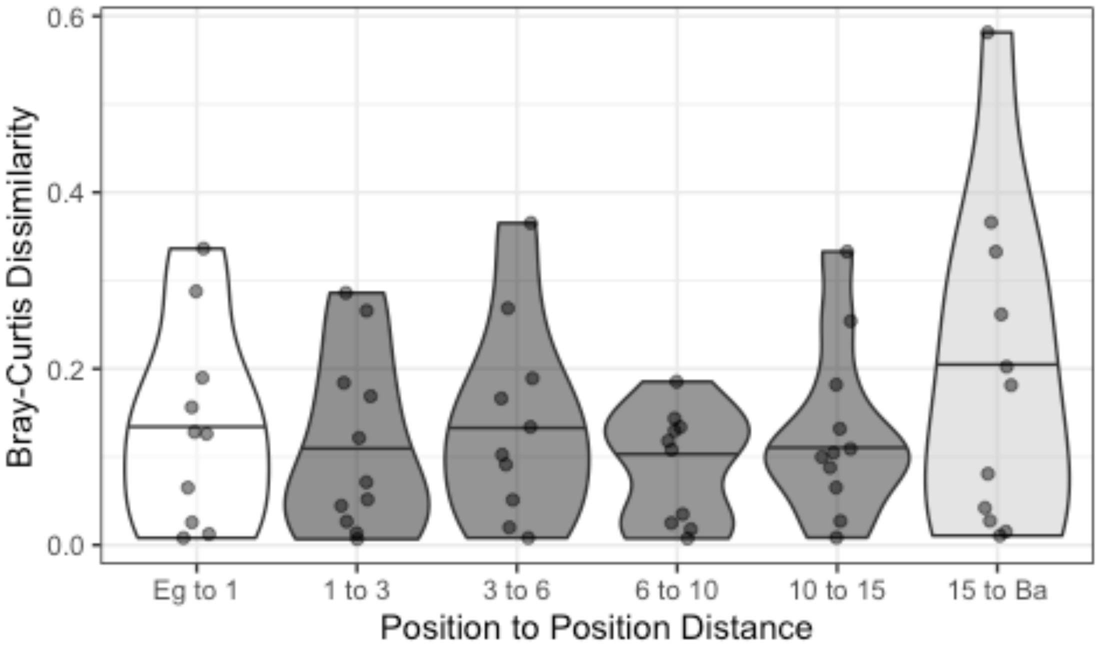

Figure S7: Ordination plots of samples along all ten fully-sampled transects from bare to eelgrass positions. Technical replicates of each biological sample are grouped as triangles. Alongshore transect samples are shown in white and labeled with distance from the contiguous eelgrass bed in meters (Al); the single sample taken above eelgrass (located 47-90 meters inside the edge of the contiguous beds) is shown in green (Eg), and that taken above bare substrate (located 16-670m outside the edge of the contiguous beds) is shown in brown (Ba). Site abbreviations are as follows: CI (Case Inlet), PG (Port Gamble), SK (Skokomish), WB (Willapa Bay).

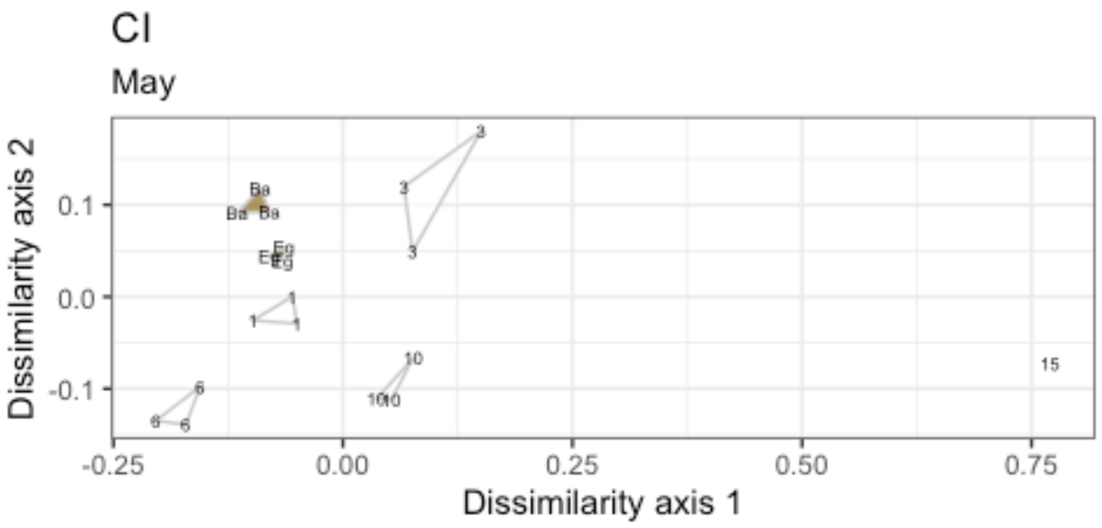

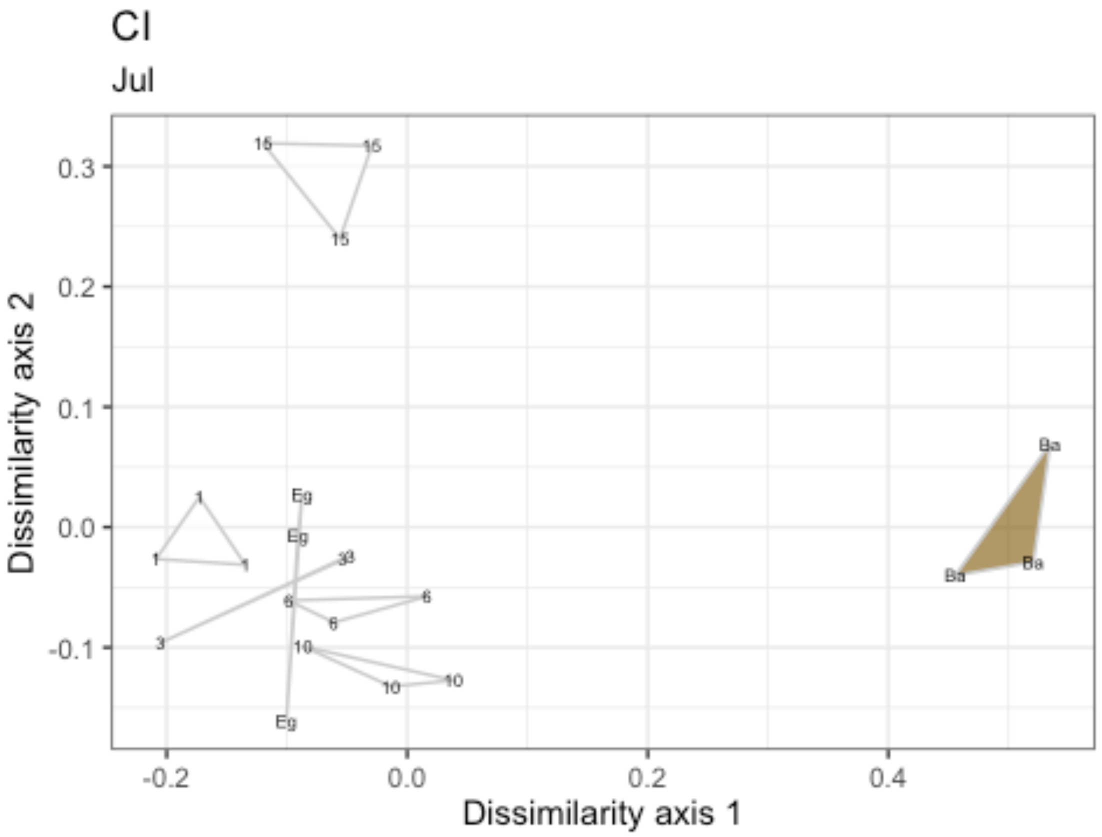

CI
Jul
Dissimilarity axis 2
Dissimilarity axis 1
0.3
0.2
0.1
0.0
-0.1
-0.2
0.0
0.2
0.4
15
1
3
6
10
Eg
Ba

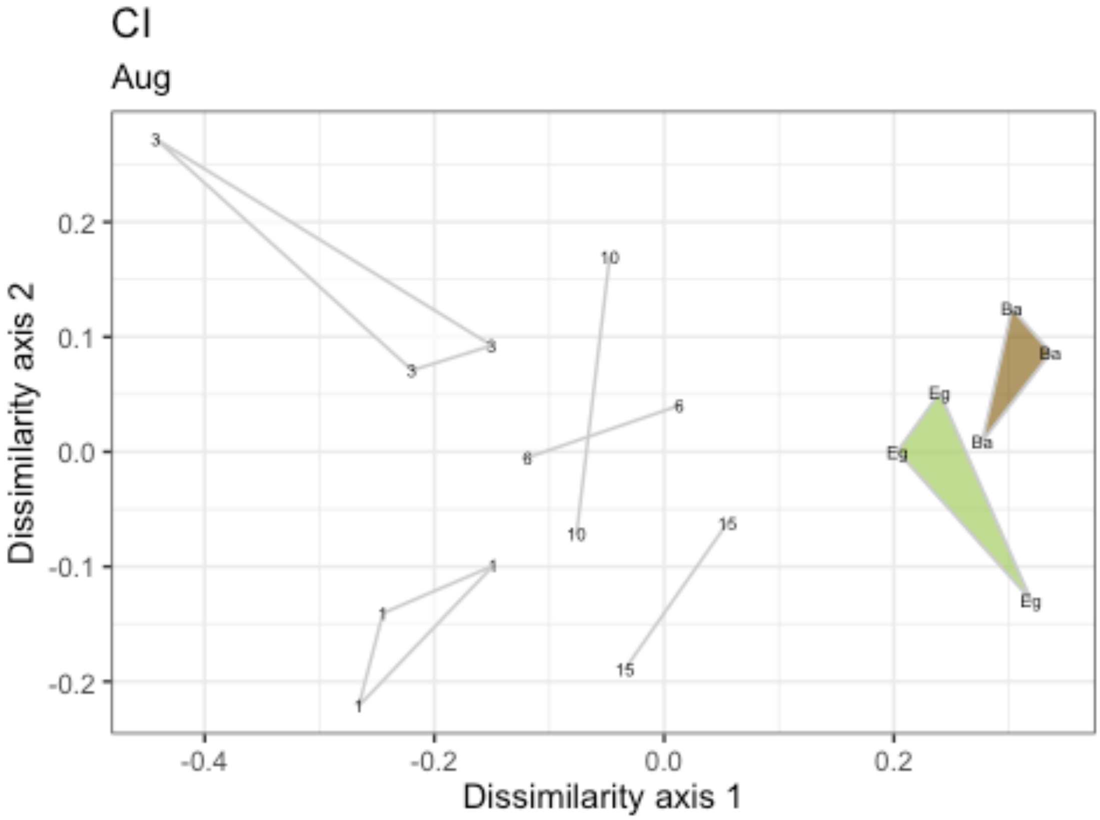

CI
Aug
Dissimilarity axis 2
Dissimilarity axis 1
0.2
0.1
0.0
-0.1
-0.2
-0.4
-0.2
0.0
0.2
3
3
3
10
10
6
6
15
15
1
1
1
Ba
Ba
Ba
Eg
Eg
Eg

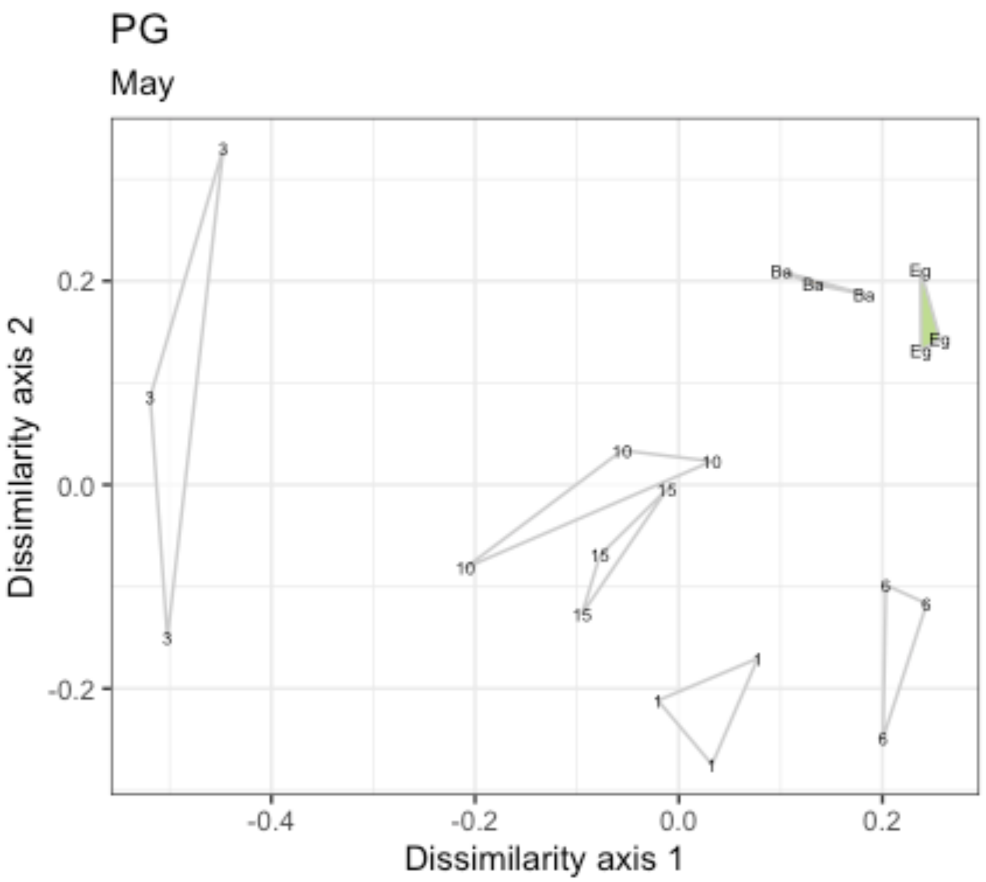

PG
May
Dissimilarity axis 2
Dissimilarity axis 1
0.2
0.0
-0.2
-0.4
-0.2
0.0
0.2
3
3
3
Ba
Ba
Ba
Eg
Eg
Eg
10
10
10
15
15
15
6
6
6
1
1
1

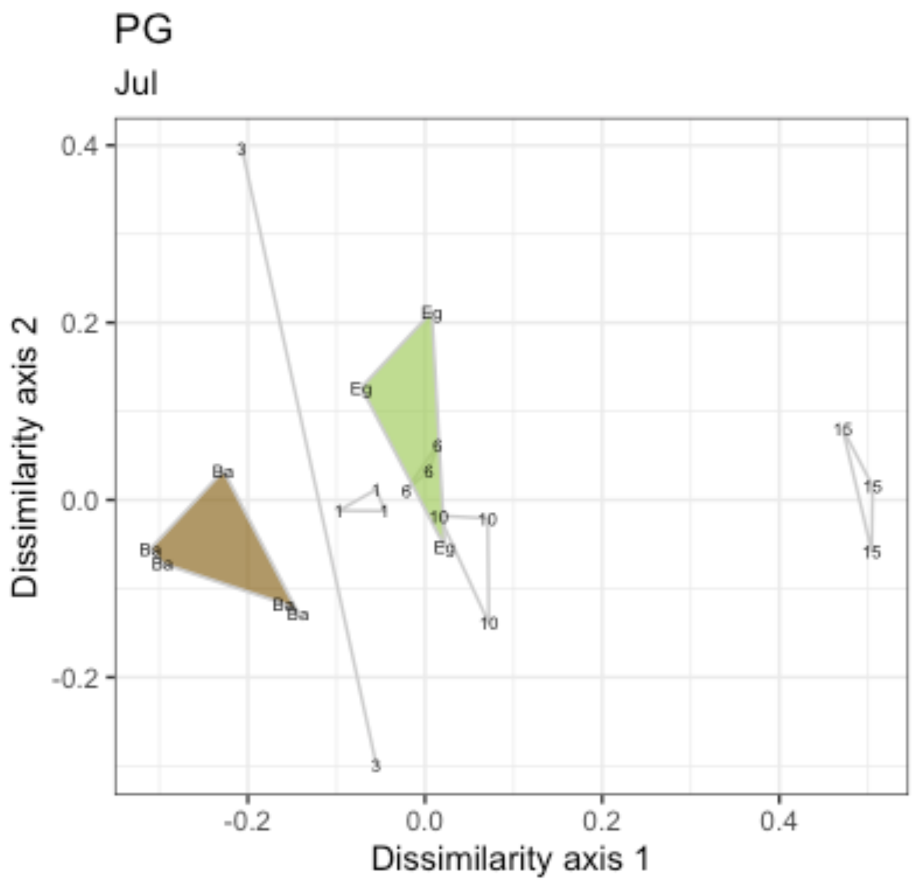
PG
Jul
Dissimilarity axis 2
Dissimilarity axis 1
0.4
0.2
0.0
-0.2
-0.2
0.0
0.2
0.4
3
Eg
Eg
6
6
6
1
1
1
10
10
10
Eg
Ba
Ba
Ba
Ba
Ba
3
15
15
15

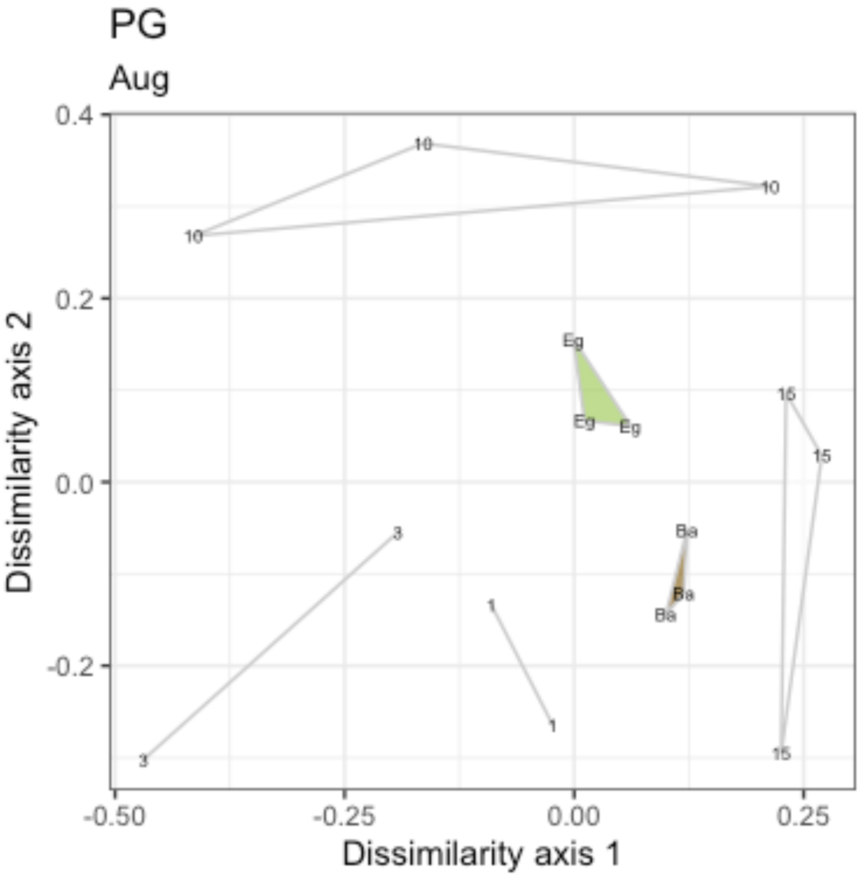

PG
Aug
Dissimilarity axis 2
Dissimilarity axis 1
0.4
0.2
0.0
-0.2
-0.50
-0.25
0.00
0.25
10
10
10
Eg
Eg
Eg
15
15
15
3
3
1
1
Ba
Ba
Ba

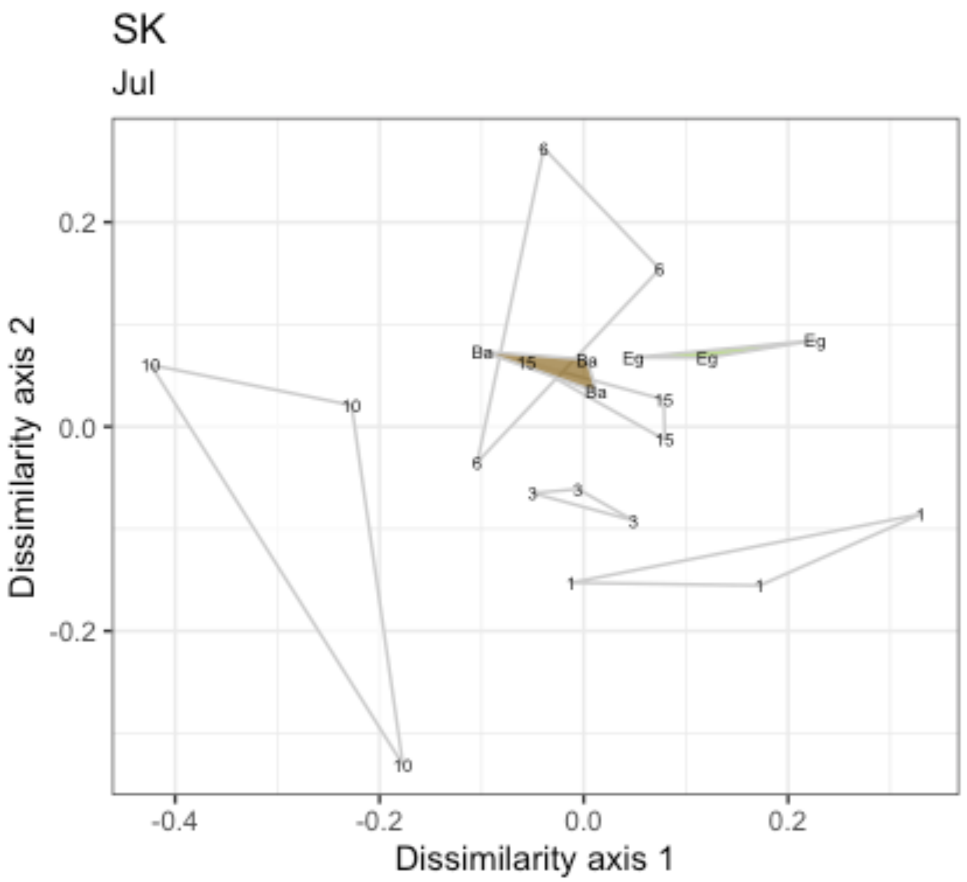
SK
Jul
Dissimilarity axis 2
Dissimilarity axis 1
0.2
0.0
-0.2
-0.4
-0.2
0.0
0.2
6
6
6
Ba
Ba
Ba
15
15
15
Eg
Eg
Eg
10
10
10
3
3
3
1
1
1

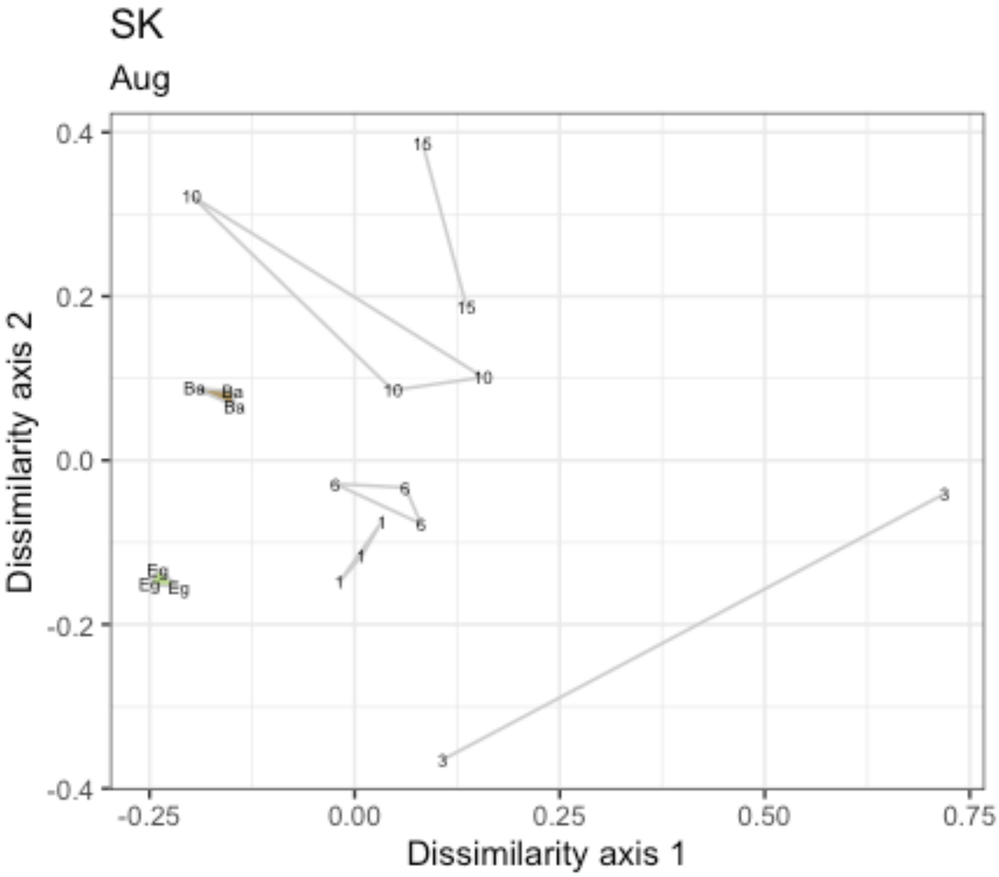
SK
Aug
Dissimilarity axis 2
Dissimilarity axis 1
0.4
0.2
0.0
-0.2
-0.4
-0.25
0.00
0.25
0.50
0.75
15
15
10
10
10
Ba
Ba
Ba
6
6
6
1
1
1
3
3
Eg
Eg
Eg

WB

May

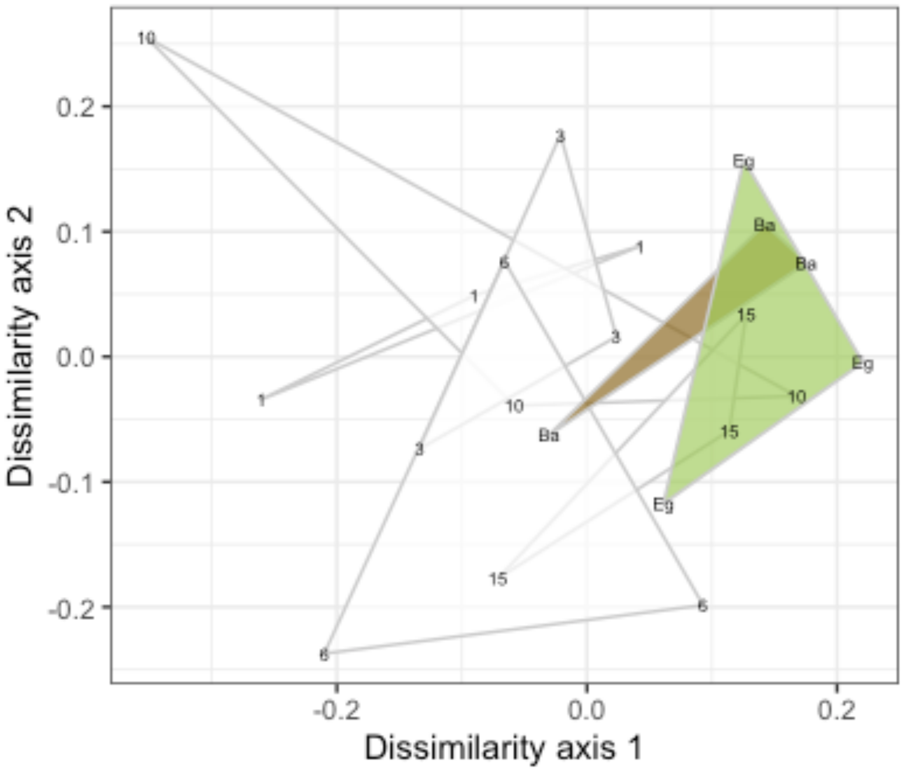

Dissimilarity axis 2
Dissimilarity axis 1
0.2
0.1
0.0
-0.1
-0.2
-0.2
0.0
0.2
10
3
Eg
Ba
1
6
Ba
1
15
3
Eg
10
10
Ba
3
15
Eg
15
6
6
1

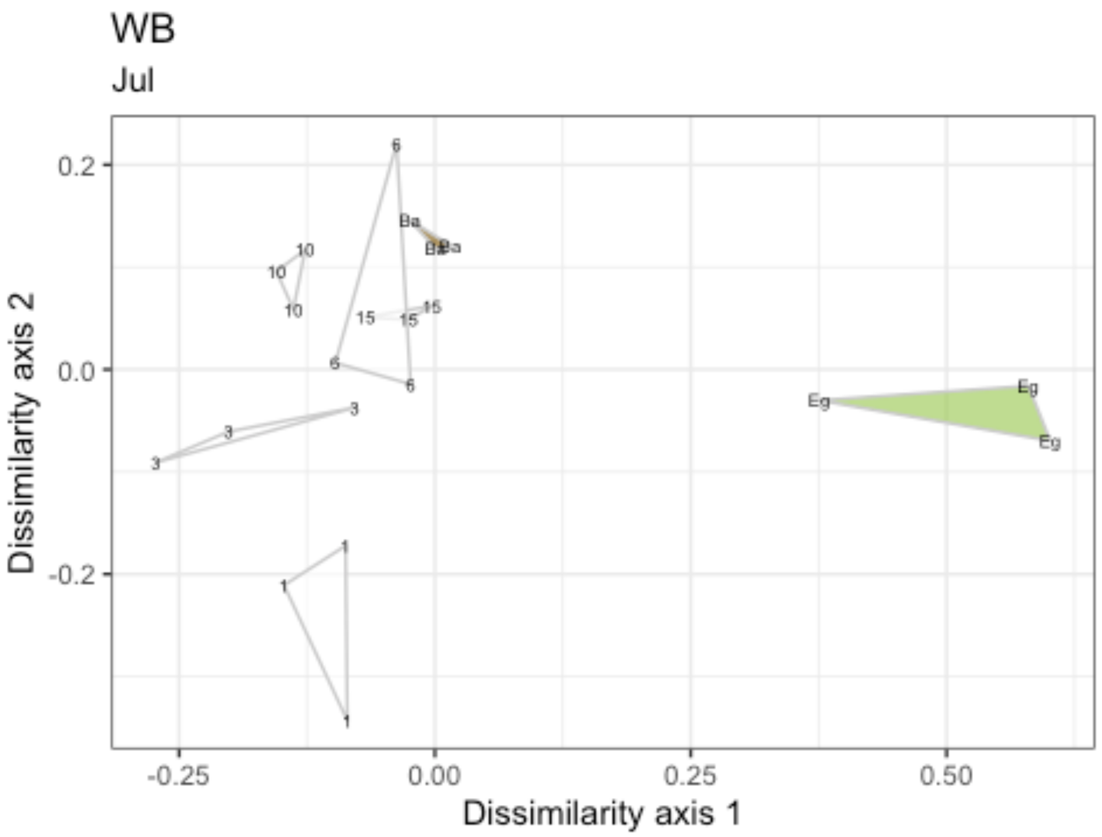

WB
Jul
Dissimilarity axis 2
Dissimilarity axis 1
0.2
0.0
-0.2
-0.25
0.00
0.25
0.50
6
Ba
10
15
3
1
Eg

Figure S9: OTU richness plotted for all sites and months at each point along the transect from eelgrass to bare substrate. Bars shown are means, boxes depict the two inner quartiles, whiskers extend to the largest value no further than 1.5 times the inter-quartile range, and data outside this range are plotted individually.

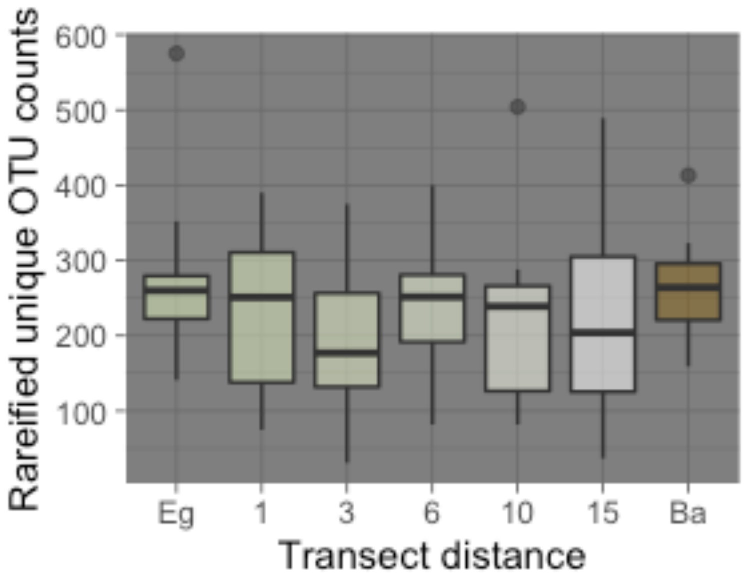

Supplement: Supplemental Information 2 [file peerj-08-8869-s002.pdf]
